# Clinical Outcomes of Arthroscopic Treatment for Triangular Fibrocartilage Complex Lesions in Adolescent Elite Athletes

**DOI:** 10.3390/jcm15010234

**Published:** 2025-12-28

**Authors:** Michele Davide Maria Lombardo, Min Cheol Chang, Loris Pegoli

**Affiliations:** 1 Istituti Clinici Zucchi, Sport Hand Surgery Unit, 20900 Monza, Italy; info@dottorlombardo.com (M.D.M.L.); info@drpegoli.com (L.P.); 2 Juventus Medical, Sport Hand Surgery Service, 10151 Torino, Italy; 3 Department of Physical Medicine and Rehabilitation, College of Medicine, Yeungnam University, Daegu 42415, Republic of Korea

**Keywords:** triangular fibrocartilage complex, injury, sports, athlete, arthroscopy, adolescent

## Abstract

**Objectives**: The triangular fibrocartilage complex (TFCC) is critical for wrist stability. The incidence of TFCC injury among adolescent athletes is rising due to increased participation in high-demand sports. Here, we investigated the therapeutic outcomes of arthroscopic TFCC repair in adolescent elite athletes. **Methods**: We retrospectively recruited 24 elite adolescent athletes (mean age 15.5 ± 1.3 years) treated arthroscopically for peripheral TFCC tears between March 2018 and January 2025. Clinical outcomes—including numeric rating scale (NRS) for pain, grip strength, wrist range of motion (ROM), DASH scores, and physical exam tests—were collected preoperatively and at 6-month follow-up. Statistical analysis included paired t-tests for continuous variables and chi-square tests for categorical comparisons. **Results**: At 6 months postoperatively, the mean NRS decreased from 6.9 ± 1.2 to 0.6 ± 0.7, grip strength increased from 26.3 ± 6.9 kg to 40.8 ± 5.6 kg, and all measured ROMs and DASH scores improved significantly. Positive findings on ballottement, Waiter’s, and piano key tests decreased significantly. There were no major complications. All athletes returned to their pre-injury sport levels. **Conclusions**: Arthroscopic peripheral TFCC repair in adolescent elite athletes is a safe, minimally invasive, and effective treatment, leading to rapid pain relief, functional recovery, and return to sport with their pre-injury sport levels.

## 1. Introduction

The TFCC (triangular fibrocartilage complex) plays a crucial role in wrist stability, particularly in the distal radioulnar joint (DRUJ). It acts as a shock absorber and stabilizer, transmitting axial forces across the wrist and allowing smooth forearm rotation [1]. Given its anatomical complexity, the TFCC is vulnerable to both traumatic injuries, which are often caused by falls on an outstretched hand or forceful wrist twisting, and degenerative changes, which result from repetitive stress over time [2]. Patients with TFCC injuries commonly report pain on the ulnar side of the wrist, which is often worsened by gripping, lifting, or rotational movements [1,2]. Some also can experience clicking or popping sensations and a sense of instability in the affected wrist [1,2].

TFCC injuries are one of the common issues among athletes, particularly in sports that require frequent wrist rotation, deviation, and axial loading [2]. Racket sports like tennis and padel, golf, hockey, basketball, and combat sports cause significant stress on the wrist, increasing the risk of TFCC damage [3]. As sport participation has increased in the pediatric and adolescent population, the incidence of TFCC injuries has concurrently increased in this age group [4]. However, TFCC injuries are often overlooked or misdiagnosed as simple tendinitis or tenosynovitis [5]. The misdiagnosis can result in inadequate treatment and unnecessary time away from sports, compromising an athlete’s career. To achieve successful treatment outcomes for TFCC injuries, it is essential to make an accurate diagnosis promptly and provide effective treatment.

For diagnosing this condition, a thorough physical examination is necessary. A physical examination for TFCC injury typically begins with palpation of the ulnar fovea sign—tenderness elicited in the soft spot between the ulnar styloid and the flexor carpi ulnaris tendon—which has about 95% sensitivity and 87% specificity for peripheral TFCC tears [6,7]. In addition, provocative maneuvers such as the press test (axial ulnar-loading pain when pushing up from a seated position), ulnocarpal stress (axial load combined with ulnar deviation and forearm rotation), and the piano key test (ulnar head volarly displaced that springs back) are used to detect pain, clicking, and instability of the distal radioulnar joint in the wrist, which can help differentiate TFCC injuries from other causes of ulnar-sided wrist pain [6,7]. These physical examination methods can aid in diagnosing TFCC injuries, but cannot provide a definitive diagnosis. Also, imaging studies, such as magnetic resonance imaging (MRI) or magnetic resonance arthrography, are known to have relatively low sensitivity and specificity for TFCC injuries [8].

Wrist arthroscopy is currently considered the gold standard for both precise diagnosis and targeted treatment. In 1986, Roth first described the basic technique of wrist arthroscopy and its application to treat ulnocarpal pain [9]. Soon thereafter, Osterman and Palmer reported their results on the arthroscopic treatment of TFCC injuries [10,11]. Since then, various surgical options and repair techniques, such as all-inside, inside-out, and outside-in sutures, have been introduced with proof of efficacy in previous studies [12]. However, most of the previous studies was limited to adult athletes. Also, although previous studies by Farr et al. have described arthroscopic TFCC treatment in pediatric and adolescent patients, there remains a lack of research specifically examining elite adolescent athletes [13,14].

In this study, we investigated the therapeutic outcomes of arthroscopic management of TFCC injuries in elite adolescent athletes using outside–inside technique.

## 2. Materials and Methods

### 2.1. Subjects

This study was retrospectively conducted. From March 2018 to January 2025, a total of 24 adolescent elite athletes (age: 15.5 ± 1.3 years [range: 13 to 18]; M:F = 9:15) were treated in our Sport Hand Surgery Center for TFCC injuries with wrist arthroscopy (Table 1). All these patients were included in our study based on the following inclusion criteria: (1) adolescents aged 12 to 18 years, (2) elite-level athletes competing in high-level regional or national competitions and participating in more than 10 h of structured training per week, (3) a confirmed diagnosis of TFCC injury, and (4) persistent ulnar-sided wrist pain. Patients with associated fractures, ligamentous injuries, or other wrist pathologies requiring additional surgical procedures were excluded. The affected side was Rt. in 13 patients and Lt. in 11 patients. The sports that the patients were engaged in were as follows: 2 boxing, 1 cycling, 1 climbing, 1 gymnastics, 1 judo, 4 kick boxing, 1 motocross, 1 ski, 2 soccer, 6 tennis, and 4 volleyball. We performed wrist arthroscopic treatment when a patient’s pain did not respond to conservative treatment for at least 6 months.

Prior to wrist arthroscopy, the diagnosis of TFCC was confirmed based on the patients’ symptoms, physical examination results, and MRI findings. Of the 24 included patients, 15 patients (62.5%) showed TFCC injury on MRI. All the included patients had only traumatic and peripheral lesions of the TFCC. One patient had a concomitant scapholunate ligament injury (grade 1 according to Geissler classification) [15].

Our study was approved by the institutional review board of a University Hospital. Written informed consent was waived owing to the retrospective nature of the study.

### 2.2. Treatment

**Figure 1:**
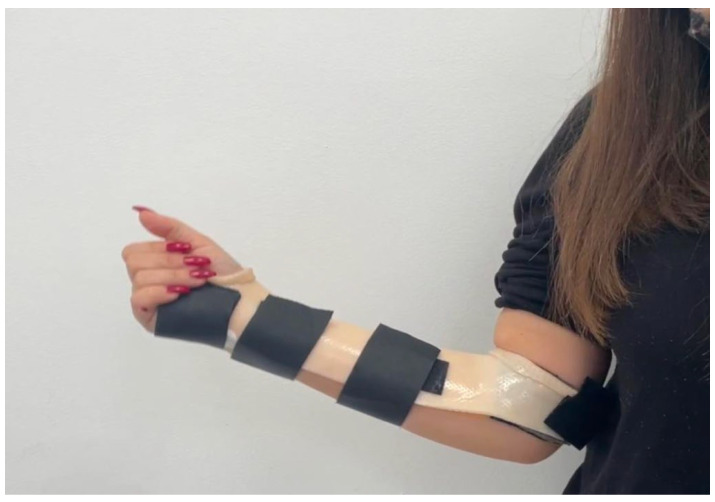
Munster splint.

After a TFCC injury was diagnosed and confirmed to meet the indications for surgery, the patients were referred directly to the hand therapist. A custom thermoplastic Muenster-style splint was fabricated for immediate postoperative use and designed to provide appropriate immobilization (Figure 1).

**Figure 2:**
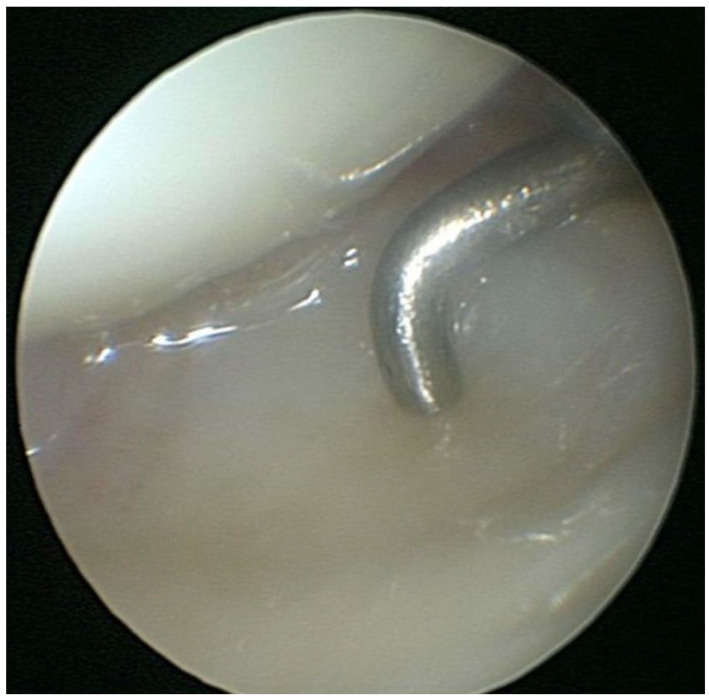
Arthroscopic view of the trampoline test.

**Figure 3:**
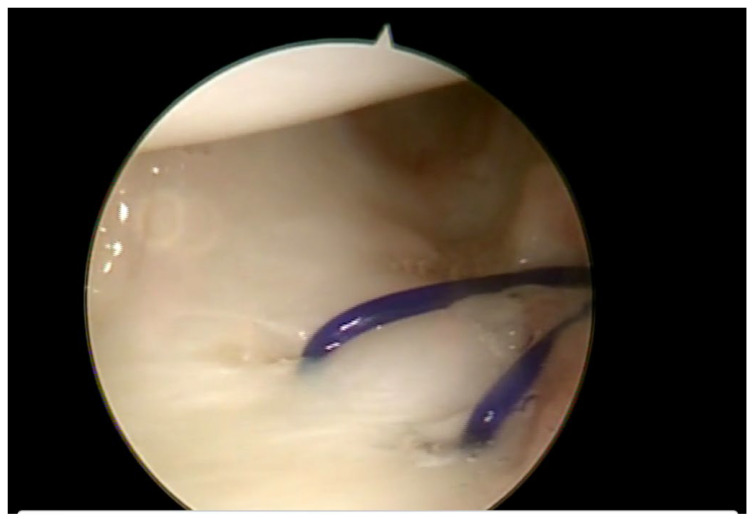
Arthroscopic view of the polydioxanone suture.

During the arthroscopic repair procedure, the patients were positioned in a supine position, and a tourniquet was inflated to 250 mmHg at the base of the upper limb. Plexus locoregional anesthesia was performed. DRUJ laxity was assessed under anesthesia before the arthroscopic procedure was initiated. Through arthroscopic portals 3–4 and 6R, the radio carpal joint was examined. A dry technique was preferred to avoid tissue imbibition. With the scope in the standard 3–4 portal, the tear was visualized in the dorsoulnar corner of the TFCC. A probe was inserted through the 6R access. Trampoline and hook tests were performed (Figure 2). Any synovitis next to the lesion was removed using a shaver. The lesion was repaired using two PDS II (polidioxanone) arthroscopic sutures (outside–inside technique) [16] (Figure 3). The technique involves placing suturing points between the capsule and the ligament. Under direct visualization with the scope through the 3–4 arthroscopic portal, debridement of the injured portion is performed, removing fibrous remnants with a shaver inserted through the 6R portal until a viable tissue plane is reached. From the same portal, an 18-gauge needle used as a shuttle is then passed to advance the PDS suture from the lower portion of the ligament into the intra-articular space. A margin of a few millimeters is left at the free edge of the TFCC. The suture is then retrieved from the intra-articular space and brought outside the joint through the 6R arthroscopic portal. The procedure is repeated with a second suture. After confirming that no nerve branches or extensor tendons are in the suture path, the knot is then tied on the articular capsule. At the end of the procedure, DRUJ stability was tested again with and without traction. The skin of the portals was sutured with an absorbable thread (4/0 Vicryl Rapide), a simple dressing was performed, and the previously made thermoplastic splint was applied.

All surgeries were performed by two expert senior surgeons. A short-term antibiotic prophylaxis was administered. After the arthroscopic treatment, the patients were followed up by the surgeons on day 15 for stitches removal, on week 4 for the modification of the splint (elbow free), and at 6 weeks, 3 months, and 6 months postoperation. The athlete was allowed to perform exercises targeting the legs, abdominals, and uninjured upper limb 1 week after surgery to maintain the physical condition he had before the injury.

**Figure 4:**
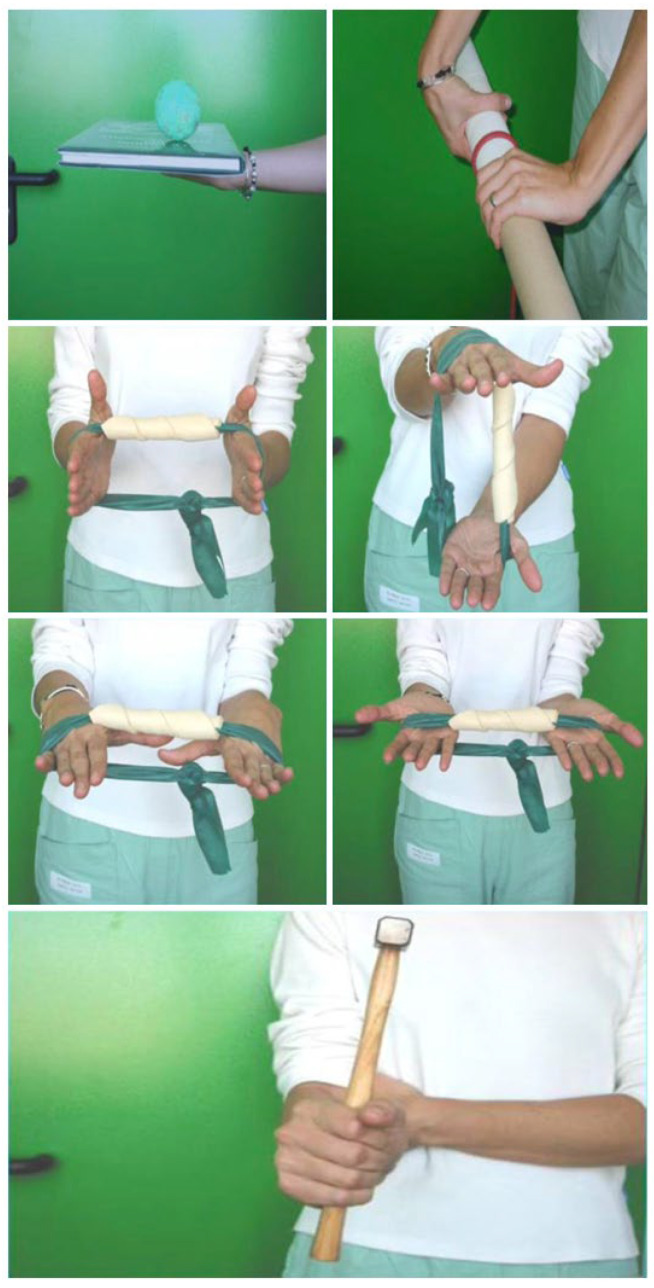
Isotonic exercises with band and strengthening protocol.

**Figure 5:**
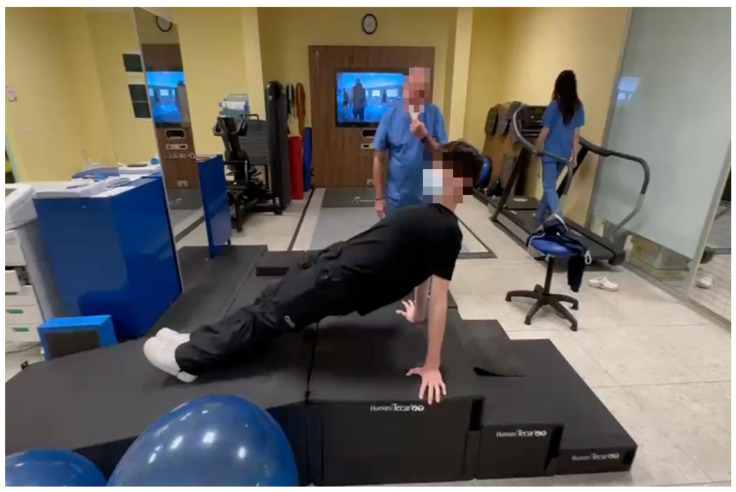
Sport reconditioning.

After 2 weeks, the dressing and stitches were removed. During the first 4 weeks, the patient was trained by the physiotherapist about edema management and hand use. At 4 weeks, the previous splint was modified, thereby freeing the elbow. The splint was gradually removed 6 weeks after surgery. During the follow-up consultation (45 days postoperation), the surgeon tested the stability of the distal radioulnar joint and the range of motion (ROM) in pronosupination, flexion, and extension. From the 8th week, passive mobilization could be introduced, especially to recover any joint motion deficits. Passive mobilization consisted of gentle pain-free passive ROM exercises of the wrist, focusing on flexion–extension and forearm pronation–supination while avoiding excessive ulnar deviation. Once the ROM was recovered, the patient could begin muscle-strengthening exercises—starting with isometric exercises of the wrist flexors, extensors, and forearm rotator muscles—followed by concentric and eccentric strengthening once full pain-free ROM was achieved (Figure 4). The surgeon allowed the athlete to resume full activity, including contact sports, 3 months after surgery and after the clinical follow-up. According to the rehabilitation protocol, the athlete was followed not only by the hand therapist but also by the musculoskeletal physiotherapist to work on the full athletic gesture, including the rest of the body, specifically on sport reconditioning (Figure 5).

### 2.3. Measurements

All patients were clinically evaluated in terms of the following: numeric rating scale (NRS); active ROM of the flexion and extension of the wrist and the pronosupination of the forearm; grip strength; and Disabilities of the Arm, Shoulder, and Hand (DASH) scores. The NRS is a validated, unidimensional self-report tool used to rate the current pain of patients on an 11-point scale from 0 (no pain) to 10 (worst pain imaginable). The DASH questionnaire is composed of 38 items that evaluate the upper extremity function and its effect on daily life [17]. The DASH score ranges from 0 to 100. Lower scores correspond to greater patient ability and less disability. Ballottement, Waiter’s, and piano key tests were conducted. All these evaluations were performed before surgery and at 6 months postoperatively.

### 2.4. Statistical Analysis

Data were statistically analyzed using IBM SPSS Statistics for Windows version 31.0 (IBM Corp., Armonk, NY, USA). Before the tests were performed, data normality was assessed using the Shapiro–Wilk test to examine the differences in continuous variables at preoperation and 6-month postoperation. Paired t-tests and chi-square tests were used to compare pre- and postoperative outcomes and categorical variables, respectively. These tests were selected because paired t-tests are appropriate for evaluating changes in continuous variables measured within the same subjects over time, whereas chi-square tests allow for comparison between categorical groups. Eight primary outcomes were analyzed. To account for the increased risk of Type I error due to multiple comparisons, we applied a Bonferroni-adjusted significance threshold of α = 0.006 (0.05/8). *p*-values < 0.006 were considered statistically significant.

## 3. Results

All 24 patients were treated surgically by arthroscopic repair (outside–inside technique). The average period from injury to diagnosis was 16.2 ± 3.3 months (range: 10.0–22.0 months). After arthroscopic repair, no major complications (infection, bleeding, and neurovascular bundle injury) were observed. Two patients had minor complications. One case presented a tenosynovitis of the extensor carpi ulnaris after the strengthening exercise because of the overload. The symptoms from the tenosynovitis resolved spontaneously when the exercises stopped. The other minor complication observed was represented by skin discomfort at the arthroscopic suture site. Because the problem resolved itself spontaneously within approximately 6 months, which was exactly the time of stitch degradation, this discomfort was probably caused by the reabsorption of the suture in polydioxanone. The wounds (arthroscopic portals) healed after an average of 9.0 ± 1.1 days (range: 7–11 days).

According to Palmer’s classification [18], the TFCC lesions found during wrist arthroscopy had the following lesions: 1C tear (3 cases), 1D tear (3 cases), and 1B tear (18 cases). The lesion of the scapholunate ligament found in one case was classified as grade 1 according to Geissler’s classification. It was treated arthroscopically with radiofrequency shrinkage. No changes in cartilage were found in any patient because of the young age.

The NRS score significantly decreased after the arthroscopic treatment from 6.9 ± 1.2 at preoperation to 0.6 ± 0.7 at 6-month follow-up after the operation (paired *t*-test, *p* < 0.001, t-statistic = 25.76; Table 1). The grip strength significantly increased after the arthroscopic treatment from 26.3 ± 6.9 kg at preoperation to 40.8 ± 5.6 kg at 6-month follow-up (paired *t*-test, *p* < 0.001, t-statistic = −25.78). The ROMs of the flexion and extension of the wrist and the pronosupination of the forearm at preoperation were 71.5° ± 8.0°, 64.9° ± 8.0°, and 132.2° ± 18.2°, respectively, and those at 6-month follow-up were 87.7° ± 4.7°, 82.6° ± 6.9°, and 175.5° ± 5.1°, respectively. All the measured ROMs significantly increased (paired *t*-test, *p* < 0.001, t-statistic = −10.43, −10.26, −14.10 in flexion, extension, and pronosupination ROMs). The DASH scores were significantly reduced at 6-month follow-up (6.0 ± 2.2) compared with those at preoperation (45.1 ± 4.4; paired *t*-test, *p* < 0.001, t-statistic = 42.74).

The number of patients testing positive on the ballottement, Waiter’s, and piano key tests decreased significantly from 24, 19, and 20 cases preoperatively to only 4, 3, and 5 cases at 6 months postoperation (chi-square test, <0.001; Table 2).

At 6 months after the operation, all the included patients reported that their use of the entire arm in daily life activities significantly improved, and they returned to their original sports at their previous levels.

## 4. Discussion

In this study, the arthroscopic repair of TFCC peripheral tears yielded excellent treatment outcomes in adolescent elite athletes with TFCC injuries. The included patients’ pain was significantly reduced (NRS: 6.9 → 0.6) 6 months after the arthroscopic operation, and all the ROMs (flexion, extension, pronation, and supination) of the wrist joint significantly increased. The function of the upper extremity also significantly increased. All the patients reported that they had functional improvement in the entire arm in daily life activities and could return to their sports at their previous levels. In our study, the transosseous suture technique commonly used for TFCC repair in adults was not applied because creating transosseous tunnels may injure the open physes and potentially compromise bone growth.

The average age of patients with wrist ligament injuries, including TFCC tears, is decreasing partly because of the increased participation in competitive sports at younger ages [19]. Functional demands on sports activities have substantially increased even among children, thereby elevating their risk of injury. Indeed, hand and wrist injuries appear more common in adolescent athletes than in adult athletes [20]. Therefore, TFCC tears in adolescent athletes should be effectively treated. If a TFCC injury is improperly treated, adolescent athletes can experience chronic wrist pain and long-term functional impairment. The timely and appropriate management of TFCC injuries in adolescent athletes promotes faster recovery, supports timely return to sports, and reduces the risk of long-term morbidity.

Various major complications, including recurrent DRUJ instability, non-union of bone, wound infection, tendon injury, and nerve injury, may occur after the open repair for TFCC injury in pediatric and adolescent patients [13,21,22]. Regarding the arthroscopic operation in pediatric and adolescent patients with TFCC injury, two previous studies demonstrated its safety and efficacy [13,14]. In 2012, Farr et al. [13] reported their therapeutic outcomes in pediatric and adolescent TFCC injury. Of the 28 TFCC tears, 14 cases underwent arthroscopic TFCC resection, and 2 cases were treated with arthroscopic inside-out repair. In one case with severe anatomical deformities, such as a Madelung deformity, arthroscopic access was limited; as such, the procedure was converted to open resection. Arthroscopic operations were successfully performed in most cases without any arthroscopy-related complications. They reported that the arthroscopic management of a TFCC tear can be a safe and effective therapeutic option for TFCC injuries in pediatric and adolescent patients. Conversely, three complications, namely, non-union of the radius, wound infection, and rupture of the extensor pollicis brevis tendon, occurred in seven cases who received open resection arthroplasty. In 2015, Farr et al. [14] reported the outcomes of an arthroscopically assisted repair of peripheral TFCC (Plamer type 1B) in 12 adolescents. After a mean follow-up of 1.3 years, the patients showed significant pain reduction, and their VAS scores improved from 7.0 to 1.7. Their functional outcome was also enhanced, with a mean postoperative DASH score of 16. At their final follow-up, grip strength did not significantly differ between the affected and non-affected sides. No major complications occurred except three cases of transient paresthesia. Therefore, arthroscopic TFCC repair is a safe and effective treatment for adolescents.

Furthermore, arthroscopic treatment can offer distinct advantages for elite adolescent athletes. Its minimally invasive nature reduces soft-tissue disruption and may help preserve the proprioceptive components of the TFCC that contribute to wrist stability and neuromuscular control, which is an essential requirement for high-level athletic performance. In addition, arthroscopy allows direct visualization of the lesion, enabling more accurate identification of tear characteristics and the application of a tailored repair technique that reflects the specific mechanical demands of each sport. Because arthroscopic procedures generally lead to less postoperative pain and swelling, rehabilitation can begin earlier and progress more aggressively, potentially facilitating a safer and faster return to pre-injury levels of performance. These combined advantages highlight the clinical relevance and suitability of arthroscopic repair for athletes who require rapid, reliable, and functionally optimized recovery.

However, in the two previous studies [13,14], arthroscopy was not conducted in athletes. Therefore, our study is the first to report the therapeutic outcomes of arthroscopic TFCC repair in adolescent elite athletes with TFCC injuries. Treating adolescent athletes with TFCC injuries can be difficult. Each athlete has individual priorities and concerns, ranging from general health and fitness for recreational athletes to the demands of athletes living as professionals [23]. Parents are also skeptical about this surgical treatment [23]. Surgeons should understand these issues and offer the appropriate treatment options at the right time [24]. The treatment for a competitive athlete with a TFCC tear aims to obtain maximal recovery and return to the pre-injury performance level. Considering our results with the absence of major complications and the excellent outcome in pain reduction and functional recovery following arthroscopic TFCC repair, we believe this approach represents a valuable alternative to open repair in pediatric athletes with TFCC injury.

In addition, our findings should be interpreted within the specific context of our study population. Because the cohort consisted exclusively of elite adolescent athletes with traumatic peripheral TFCC tears, the results may not be generalizable to non-elite adolescents, individuals with degenerative or central TFCC lesions, or patients treated in community practice settings. Differences in physical demands, baseline wrist function, and access to specialized rehabilitation may also limit the broader applicability of these findings.

In conclusion, arthroscopic peripheral TFCC repair in adolescent elite athletes yielded an excellent therapeutic outcome characterized by substantial pain relief and significant improvement in grip strength, ROM of the wrist joint, and upper limb function. All the included patients showed a high functional return to pre-injury sports activity and no major complications. We believe that arthroscopic TFCC repair is a safe, effective, and minimally invasive alternative to open repair in adolescent athletes, facilitating rapid recovery and return to sport with pre-injury sports activity. The applicability of these results is limited to elite adolescent athletes with traumatic peripheral TFCC injuries. Therefore, the findings should not be generalized to non-elite adolescents, patients with degenerative or central TFCC tears, or individuals treated outside specialized sports-medicine settings. Our study is limited by its retrospective nature and the relatively small number of patients. Because this study was retrospective and involved a relatively small sample size, selection bias may have influenced the findings. Also, the follow-up duration was limited to 6 months. Although this period provided valuable early clinical outcomes, long-term follow-up would strengthen the robustness of the findings. The relatively short follow-up period may not be sufficient to fully evaluate long-term outcomes, including the risk of recurrence and the development of future wrist-related problems. In addition, our study did not include a control or comparison group, which prevents us from drawing definitive conclusions regarding the superiority of arthroscopic repair over other treatment options. Lastly, our study is susceptible to observer bias, as the surgeons who performed the procedures also conducted the postoperative assessments, and no blinding was implemented. As such, future studies should be performed to address our limitations.

## Figures and Tables

**Table 1:** Measurement outcomes at preoperation and 6-month postoperation.

	Preoperation	Postoperation	*p*-Value	T-Statistic
NRS	6.9 ± 1.2	0.6 ± 0.7	<0.001 *	25.76
Grip strength (kg)	26.3 ± 6.9	40.8 ± 5.6	<0.001 *	−25.78
ROM (flexion; °)	71.5 ± 8.0	87.7 ± 4.7	<0.001 *	−10.43
ROM (extension; °)	64.9 ± 8.0	82.6 ± 6.9	<0.001 *	−10.26
ROM (pronosupination; °)	132.2 ± 18.2	175.5 ± 5.1	<0.001 *	−14.10
DASH	45.1 ± 4.4	6.0 ± 2.2	<0.001 *	42.74

Abbreviations: NRS, numeric rating scale; ROM, range of motion; DASH, Disabilities of the Arm, Shoulder, and Hand. * <0.05, statistical significance.

**Table 2:** Results of ballottement test, Waiter’s test, and piano key test.

	Preoperation	Postoperation	*p*-Value	χ^2^ Value
Ballottement test (+/−), n	24/0	4/20	<0.001 *	34.29
Waiter’s test (+/−), n	19/5	3/21	<0.001 *	21.48
Piano key test (+/−), n	20/4	5/19	<0.001 *	18.78

* <0.05, statistical significance.

## Data Availability

Data are contained within the article.

## Abbreviations

NRS	Numeric Rating Scale
ROM	Range of Motion
DASH	Disabilities of the Arm, Shoulder, and Hand
VAS	Visual Analog Scale
TFCC	Triangular Fibrocartilage Complex
DRUJ	Distal Radioulnar Joint
MRI	Magnetic Resonance Imaging
